# Genome Sequence of *Vibrio* sp. Strain CCB-PB317, Isolated from a Malaysian Mangrove, and Its Arsenic Resistance Mechanisms

**DOI:** 10.1128/mra.01000-22

**Published:** 2023-01-04

**Authors:** Noorizan Miswan, Nyok-Sean Lau, Nor Azura Azami, Go Furusawa

**Affiliations:** a Centre for Chemical Biology, Universiti Sains Malaysia, Bayan Lepas, Penang, Malaysia; Wellesley College

## Abstract

*Vibrio* sp. strain CCB-PB317 with potential arsenic detoxification was isolated from a mangrove in Pulau Betong, Malaysia. Here, we report a draft genome sequence of strain CCB-PB317, which comprised 5,157,574 bp with a G+C content of 44.9%. The genome contains genes related to an arsenic resistance system coupled with glycolytic metabolism.

## ANNOUNCEMENT

The mangrove sediment (approximately 1 cm depth) in Pulau Betong, Malaysia, (5°20′17.016″N, 100°11′37.5″E) was sampled using a sterilized 50-mL Falcon tube. One gram of the sediment was suspended in 10 mL of sterilized 2.4% artificial seawater with 10 mM HEPES (pH 7.6). Strain CCB-PB317 was isolated based on the method described by Dinesh et al. (1) using a high-nutrient artificial seawater agar medium (H-ASWM) (2). The isolate was purified two times by single-colony isolation. The strain was maintained on marine agar (MA) at 30°C. The 16S rRNA gene of strain CCB-PB317 was amplified using the primer pair 27F/1492R by colony PCR. The sequence analysis of CCB-PB317 showed 99.86% similarity to Vibrio alginolyticus (GenBank CP006718) based on the EzBioCloud database (3).

Strain CCB-PB317 cells cultured with 10 mL of H-ASWM broth were used for the following DNA extraction. DNA extraction was carried out according to the method described by Sokolov (4) with slight modifications. DNA was extracted by the chloroform extraction method with KCl to remove polysaccharides and proteins (4). The extracted DNA was subjected to AMPure SRPI beads to purify large-size DNA fragments (5). Whole-genome sequencing was carried out using the Nanopore and Illumina platforms from the same DNA extraction according to the manufacturer’s instructions. For Nanopore library preparation, approximately 400 ng of DNA measured by Qubit was fragmented using the Nanopore rapid barcoding kit without size selection. The sample was sequenced with a R.9.4.1 flow cell on a Nanopore MinION device. Guppy v4.4.1 was used to extract the fast5 file in high-accuracy mode. For Illumina library preparation, approximately 100 ng of DNA was fragmented to 350 bp using a Bioruptor system before being processed with the New England BioLabs (NEB) Ultra II library preparation kit. The sample was sequenced on an Illumina NovaSeq6000 instrument, generating approximately 1 Gb of paired-end 150-bp data. Raw Nanopore reads were filtered with NanoFilt v2.6.0 to retain reads that were longer than 2,000 bp with scores of 7 or higher, yielding 46,174 clean reads with an *N*_50_ length of 4.263 Kb totaling 154.32 Mb. Raw Illumina reads were trimmed with fastp v0.21 to remove low-quality bases and adapter sequences. The filtered Nanopore data were then used in combination with the Illumina reads for hybrid assembly using Unicycler v0.5.0 with default settings (6). Contigs shorter than 500 bp were removed. The assembled genome of CCB-PB317 comprised 21 contigs with a total of 5,157,574 bp in length and a 44.9% G+C content. The *N*_50_ value of the assembly was 183,1286 bp. The genome annotation was carried out using the NCBI Prokaryotic Genome Annotation Pipeline (PGAP) v6.1 with GeneMarkS-2+ (7). The assembled genome annotation and functional assignment resulted in a total of 4,813 genes, 4,631 proteins, 148 RNAs (27 rRNAs, 117 tRNAs, and 4 other RNAs), and 34 pseudogenes. In addition, genes encoding a major facilitator superfamily (MFS) protein, ArsJ, and ArsJ-associated glyceraldehyde-3-phosphate dehydrogenase (GAPDH) involved in the pentavalent arsenic [As(V)] resistance (8) were discovered in the genome, indicating that strain CCB-PB317 might play an important role in the arsenic biogeocycle in the marine environment.

### Data availability.

This whole-genome project has been deposited in DDBJ/EMBL/GenBank under the assembly accession number JALGNU000000000, BioProject accession number PRJNA818689, and BioSample accession number SAMN26874050. Raw sequences have been deposited under SRA accession numbers SRR21422415 and SRR21422416.
